# Expanding the phenotypic and imaging spectrum of *GFPT1*-related congenital myasthenic syndromes: a Brazilian case series

**DOI:** 10.3389/fneur.2025.1683325

**Published:** 2025-11-14

**Authors:** Antonio Edvan Camelo-Filho, Gustavo Rodrigues Ferreira Gomes, Pedro Lucas Grangeiro Sá Barreto Lima, Vitória Maria Torres Peixoto, Tamiris Mariano, Ellen Mourão Soares Lopes, Raquel Diógenes Alencar, Paulo Ribeiro Nóbrega, André Luiz Santos Pessoa

**Affiliations:** 1 Division of Neurology, Department of Clinical Medicine, Universidade Federal do Ceará, Fortaleza, Ceará, Brazil; 2 Center of Health Sciences, Universidade Estadual do Ceará, Fortaleza, Ceará, Brazil; 3 Hospital Infantil Albert Sabin, Fortaleza, Ceará, Brazil

**Keywords:** congenital myasthenia syndromes (CMS), GFPT1, muscle ultrasound, neuromuscular disorder, Heckmatt scale

## Abstract

**Introduction:**

GFPT1-related congenital myasthenic syndrome (CMS) is a rare, autosomal recessive disorder that impairs neuromuscular transmission due to defective glycosylation of the neuromuscular junction. While typically presenting with limb-girdle weakness, tubular aggregates on biopsy, and a favorable response to acetylcholinesterase inhibitors, the full phenotypic and imaging spectrum remains incompletely defined.

**Methods:**

We evaluated five Brazilian patients from two unrelated families, all with pathogenic variants in homozygosity in *GFPT1* c.41G>A (p.Arg14Gln). Clinical, electrophysiological, and imaging assessments included nerve conduction studies, electromyography, repetitive nerve stimulation (RNS), and muscle ultrasound graded using the modified Heckmatt scale. Functional severity was estimated using the Myasthenia Gravis Foundation of America (MGFA) classification.

**Results:**

All patients showed early-onset proximal weakness, distal lower limb weakness, and frequent falls. One patient exhibited atypical features, including neonatal onset epilepsy, and cognitive impairment. RNS revealedmarked decrements in proximal upper-limb muscles (deltoid 43.6%, trapezius 37.3%) and in the distal lower-limb tibialis anterior (36.5%), consistent with foot dorsiflexion weakness. Muscle ultrasound revealed varying degrees of myopathic echogenicity. A strong positive correlation was found between MGFA severity and mean Heckmatt score (*p* = 0.028), suggesting alignment between functional severity and muscle structural changes.

**Discussion:**

Our findings expand the clinical spectrum of GFPT1-CMS to include possible central nervous system involvement and demonstrate the value of integrating electrophysiology and muscle ultrasound into diagnostic evaluation. Muscle ultrasound may serve as a structural biomarker for phenotypic stratification in CMS, and distal involvement—particularly foot dorsiflexion weakness—represents an additional diagnostic clue for GFPT1-CMS.

## 1 Introduction

Congenital myasthenic syndromes (CMS) are heterogeneous inherited disorders affecting neuromuscular transmission, leading to a broad spectrum of clinical manifestations, including abnormal fatigability and temporary or persistent weakness in extraocular, facial, oral, trunk, respiratory, or limb muscles (1–3).

CMS are caused by pathogenic variants in multiple genes, most of which are inherited in an autosomal recessive manner (4). The clinical response to treatment varies across CMS subtypes, reflecting the distinct molecular mechanisms underlying each genetic defect (5, 6). It is also important to note that the term congenital may be misleading in some cases, as symptoms can first appear later in life, including in adulthood (4, 7, 8).

The glutamine-fructose-6-phosphate transaminase 1 (*GFPT1*) gene encodes an enzyme involved in protein glycosylation pathways, leading to a limb-girdle muscle weakness pattern characterized by tubular aggregates in muscle biopsies (9, 10). This CMS is characterized by a favorable response to acetylcholinesterase inhibitors (AChEIs), and its prevalence is estimated to account for approximately 0.5 to 2% of all CMS cases (11, 12).

In this study, we report five individuals from two unrelated Brazilian families with *GFPT1*-CMS caused by homozygous pathogenic variants in *GFPT1*, specifically the previously reported missense variant c.41G>A (p.Arg14Gln) (13) We describe an integrated assessment with muscle ultrasound, nerve conduction studies (NCS), and electromyography (EMG), highlighting the clinical and imaging spectrum, intrafamilial variability in disease severity, and possible novel features such as epilepsy.

## 2 Methods

### 2.1 Participants

Based on medical records, five patients with *GFPT1*-CMS were identified from a specialized outpatient clinic for neuromuscular diseases in Brazil. All patients had a confirmed genetic diagnosis of *GFPT1*-CMS and underwent an electrophysiological workup, including NCS, EMG, and muscle ultrasound. All patients underwent detailed clinical examinations, including assessments of muscle strength, respiratory function, and bulbar symptoms. MGFA (Myasthenia Gravis Foundation of America) scores (14) were collected, and motor function was assessed using the Medical Research Council (MRC) scale. Responses to treatments, including pyridostigmine, beta2-agonists, and other therapeutic interventions, were documented through subjective assessment of symptoms. The study was approved by the Ethics Committee of the Albert Sabin Hospital under protocol number 4.756.565. Written informed consent was obtained from all participants before enrollment.

### 2.2 Genetics

All patients had a confirmed genetic diagnosis of *GFPT1*-CMS. DNA was extracted from oral swab samples, and whole exome sequencing (WES) was performed using the NovaSeq Illumina platform at Mendelics Genomic Analysis (São Paulo, Brazil), a clinical laboratory improvement amendments (CLIA)-certified laboratory. Variants were classified according to the American College of Medical Genetics (ACMG) guidelines (15). We identified the variant c.41G>A (p.Arg14Gln), previously reported in the literature as pathogenic and associated with *GFPT1*-CMS (13). Segregation analysis could not be performed.

### 2.3 Ultrasound and electrophysiology

An experienced neurosonologist (A.E.C.) conducted B-mode muscle ultrasound using high-resolution linear probes (12–15 MHz, LOGIQ E GE Healthcare, Chicago, IL, USA). Muscle imaging was evaluated using a visual four-point grading system based on a modified Heckmatt scale (16). This scale classifies muscle echogenicity and architecture into four grades (17): Grade 1 indicates normal muscle structure with clearly visible intramuscular septa and normal background echogenicity. Grade 2 reflects mildly increased echogenicity, with septa still well-defined. Grade 3 corresponds to moderately increased echogenicity and reduced visibility of the septa. Grade 4 represents severely abnormal muscle architecture, characterized by markedly increased echogenicity and absence of distinguishable intramuscular septa (17).

Additionally, patients underwent NCS/EMG testing under standard conditions using a Litebox system (Neurosoft, Ivanovo, Russia). The NCS protocol included motor conduction studies of the median, ulnar, fibular, and tibial nerves, as well as antidromic sensory studies of the median, ulnar, sural, and radial nerves. Repetitive nerve stimulation (RNS) was performed bilaterally at 2 Hertz (Hz) in the deltoid muscle and at 3 Hz in the nasal, abductor pollicis brevis, abductor digiti minimi, and tibialis anterior muscles, with an abnormal response defined as a decrement in compound muscle action potential (CMAP) amplitude greater than 10% between the first and fourth responses. EMG was performed with concentric needle electrodes in the deltoid, biceps brachii, vastus lateralis, tibialis anterior, and medial gastrocnemius muscles to assess myopathic changes.

### 2.4 Statistics

Statistical analyses were performed using StatDisk, version 13 (New Jersey, USA). Exploratory analyses were conducted to assess potential associations between NCS, EMG, muscle echogenicity, and clinical severity measures. To evaluate the relationship between functional severity and structural muscle involvement, we applied a modified grading scheme based on the Myasthenia Gravis Foundation of America (MGFA) clinical classification (14). The MGFA classes were converted into ordinal scores to enable quantitative analysis, with MGFA IIa, IIIa, and IVa corresponding to scores of 1, 2, and 3, respectively. Muscle structural involvement was quantified using the mean Heckmatt score (16), calculated as the average echogenicity grade across five muscles (deltoid, biceps, vastus lateralis, tibialis anterior, and gastrocnemius medialis), as assessed by B-mode muscle ultrasound using the modified Heckmatt scale. A Spearman correlation was used to test the association between MGFA score and mean Heckmatt score, given the ordinal nature of MGFA categories and the small sample size. A *p*-value < 0.05 was considered indicative of statistical significance.

## 3 Results

**Figure 1:**
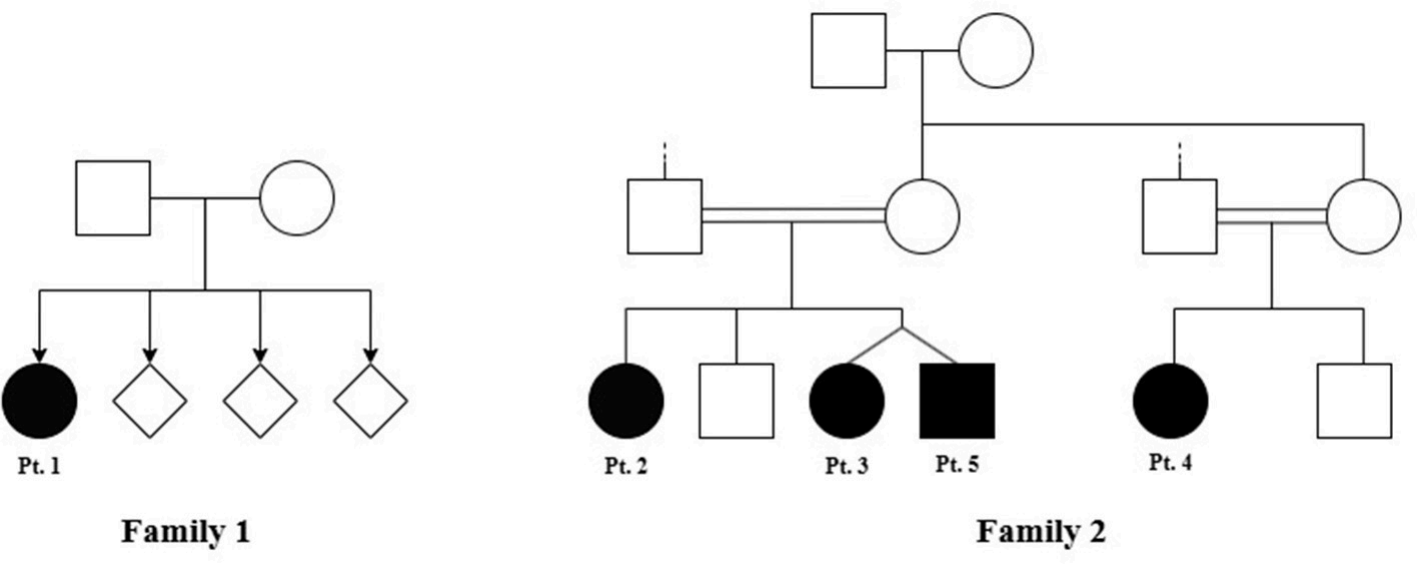
Pedigrees of families with GFPT1-CMS. Filled symbols indicate affected individuals, open symbols indicate unaffected individuals, and diamonds represent individuals of unspecified sex. The probands are labeled Pt. 1–Pt. 5. In Family 1, only Pt. 1 is affected. In Family 2, Pt. 2, Pt. 3 (twin), Pt. 4, and Pt. 5 (twin) are affected.

Clinical, demographic, and genetic features are summarized in Table 1, and the family pedigree is shown in Figure 1. All five patients carried the homozygous c.41G>A (p.Arg14Gln**)** variant in *GFPT1*. Patient one belongs to family one and has three unaffected siblings. Family two includes Patients 2, 3, 4, and 5. Patients 2, 3, and 5 are siblings, with patients 3 and 5 being twins, and patient 4 is their cousin. Symptom onset ranged from neonatal to 10 years of age, with a mean diagnostic delay of approximately 8.2 years (range: 3–16 years).

**Table 1:** Clinical, demographic, and genetic characteristics of patients with GFPT1-CMS.

***GFPT1* variants**	**Case 1**	**Case 2**	**Case 3**	**Case 4**	**Case 5**
	**c.41G**>**A (p.Arg14Gln)**	**c.41G**>**A (p.Arg14Gln)**	**c.41G**>**A (p.Arg14Gln)**	**c.41G**>**A (p.Arg14Gln)**	**c.41G**>**A (p.Arg14Gln)**
Gender	F	F	F	F	M
Age at evaluation (y)	23	13	10	20	10
Age of diagnosis (y)	20	9	6	20	6
Age of initial symptoms (y)	4	3	3	10	Neonatal
Delay in diagnosis (y)	16	6	3	10	6
Development	Age-appropriate; early-onset falls	Age-appropriate; early-onset falls	Age-appropriate; early-onset falls	Age-appropriate; early-onset falls	Motor and cognitive delay
Proximal weakness	Yes	Yes	Yes	Yes	Yes
Foot dorsiflexion weakness	Yes	Yes	Yes	Yes	Yes
MGFA	IIa	IIa	IIIa	IIa	IVa
Neonatal hypotonia	No	No	No	No	Yes
Respiratory compromise	No	No	No	No	No
Age of loss of ambulation	–	–	–	–	8
Dysphagia	No	No	No	No	Yes
Facial/ocular findings	No	No	No	No	No
Muscle biopsy	Subsarcolemmal Tubular aggregates	N/A	N/A	N/A	N/A
Response to pyridostigmine	Yes	Yes	Yes	Yes	Yes
Other clinical features	–	–	–	–	Intellectual disability psychiatric symptoms epilepsy

None of the patients exhibited facial or ocular clinical signs or symptoms. The phenotypic severity of muscle weakness in this cohort ranged from mild proximal limb involvement with preserved functional mobility (MGFA IIa) to a severe generalized presentation with early loss of ambulation (MGFA IVa), all occurring in the absence of ptosis or bulbar involvement (see Table 1). All patients also exhibited distal lower-limb weakness, represented clinically by foot dorsiflexion impairment. Neonatal hypotonia was observed in only one patient (Pt 5), who also had the earliest age of onset (neonatal) and the most severe disease course, being the only individual who experienced loss of ambulation at age 8. Patient 5 showed additional features not typically described in *GFPT1*-CMS, including intellectual disability, psychiatric symptoms, and epilepsy.

Patient 5 had epilepsy characterized by focal seizures that occasionally evolved into generalized tonic-clonic seizures. The patient's electroencephalogram (EEG) showed diffuse background slowing with epileptiform discharges. Treatment with carbamazepine resulted in good seizure control. However, the patient remains severely dependent on others for daily activities. Brain imaging was normal in all patients. No patients reported respiratory compromise. All patients demonstrated clinical improvement with pyridostigmine. However, the extent of response could not be formally quantified, as standardized evaluation tools such as MG-ADL, QMG, or 6MWT were not applied. Patients reported subjective improvement, notably reduced fatigue, greater agility, and increased speed, although objective changes at neurological examination did not accompany these benefits.”

**Figure 2:**
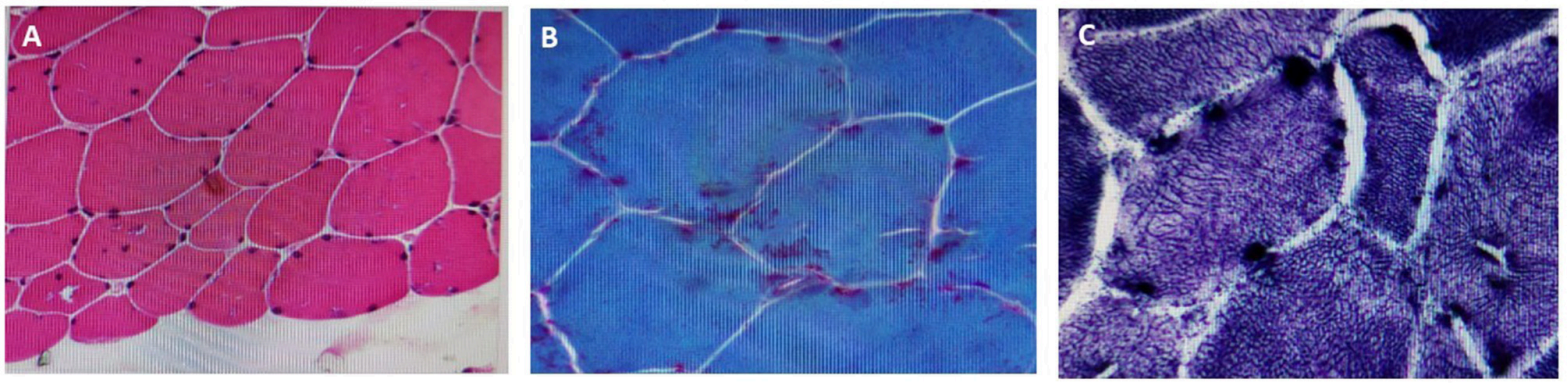
Muscle biopsy findings in patient 1 with GFPT1-related CMS. **(A)** Hematoxylin and eosin (H&E) staining showing variation in fiber size with scattered atrophic fibers. **(B)** Modified Gomori trichrome staining revealing vacuolated fibers and tubular aggregates. **(C)** Electron microscopy demonstrating numerous subsarcolemmal tubular aggregates.

Muscle biopsy was only performed in Case 1, revealing a large amount of subsarcolemmal tubular aggregates on electron microscopy (Figure 2). Motor and sensory conduction NCS were unremarkable for patients 1–4. Patient 5 did not cooperate with the NCS/EMG evaluation RNS consistently demonstrated a decremental response pattern (Table 2). The most pronounced decrements were observed in the proximal upper-limb muscles, with mean amplitude reductions of 43.6% (range 39.0–45.7) in the deltoid and 37.3% (28.5–48.8) in the trapezius. Notably, the distal lower-limb tibialis anterior was also severely affected, showing a mean decrement of 36.5% (21.7–61.7), highlighting the consistent involvement of foot dorsiflexion. In contrast, distal upper-limb muscles were only mildly impaired, with mean decrements of 13.8% (8.8–17.4) in the abductor pollicis brevis and 11.3% (7.6–14.1) in the abductor digiti minimi. Facial territory (nasalis muscle) was spared, with no decrement found. EMG revealed a chronic proximal and distal myopathy affecting all four limbs, showing short-duration, low-amplitude motor unit potential and early recruitment in patients 1–4.

**Table 2:** RNS decremental responses by muscle in patients with GFPT1-CMS.

**Patient**	**Nasalis**	**Trapezius**	**Muscles**			**Tibialis anterior**
			**Deltoid**	**Abductor pollicis brevis**	**Abductor digiti minimi**	
1	2.15	**35.05**	**45.25**	**17.35**	7.55	**25.2**
2	0.6	**48.8**	**45.7**	**15.8**	**11.4**	**61.7**
3	5.5	**36.7**	**39**	8.8	**12.2**	**21.7**
4	2.9	**28.5**	**44.4**	**13.2**	**14.1**	**37.3**
5	N/A	N/A	N/A	N/A	N/A	N/A

Repetitive nerve stimulation (RNS) results in patients with GFPT1-CMS. The table shows the percentage decrement in compound muscle action potential (CMAP) amplitude at 3 Hz across six muscles, calculated from the first to the fourth potential on the right side of the body. Bold numbers indicate abnormal decrements greater than 10%. Deltoid, trapezius and the tibialis anterior displayed the most pronounced decrements. The nasalis muscle was largely spared. Patient 5 did not undergo RNS due to lack of cooperation. RNS, Repetitive nerve stimulation; CMAP, Compound muscle action potential; GFPT1-CMS, Glutamine-fructose-6-phosphate transaminase 1-related congenital myasthenic syndrome.

**Figure 3:**
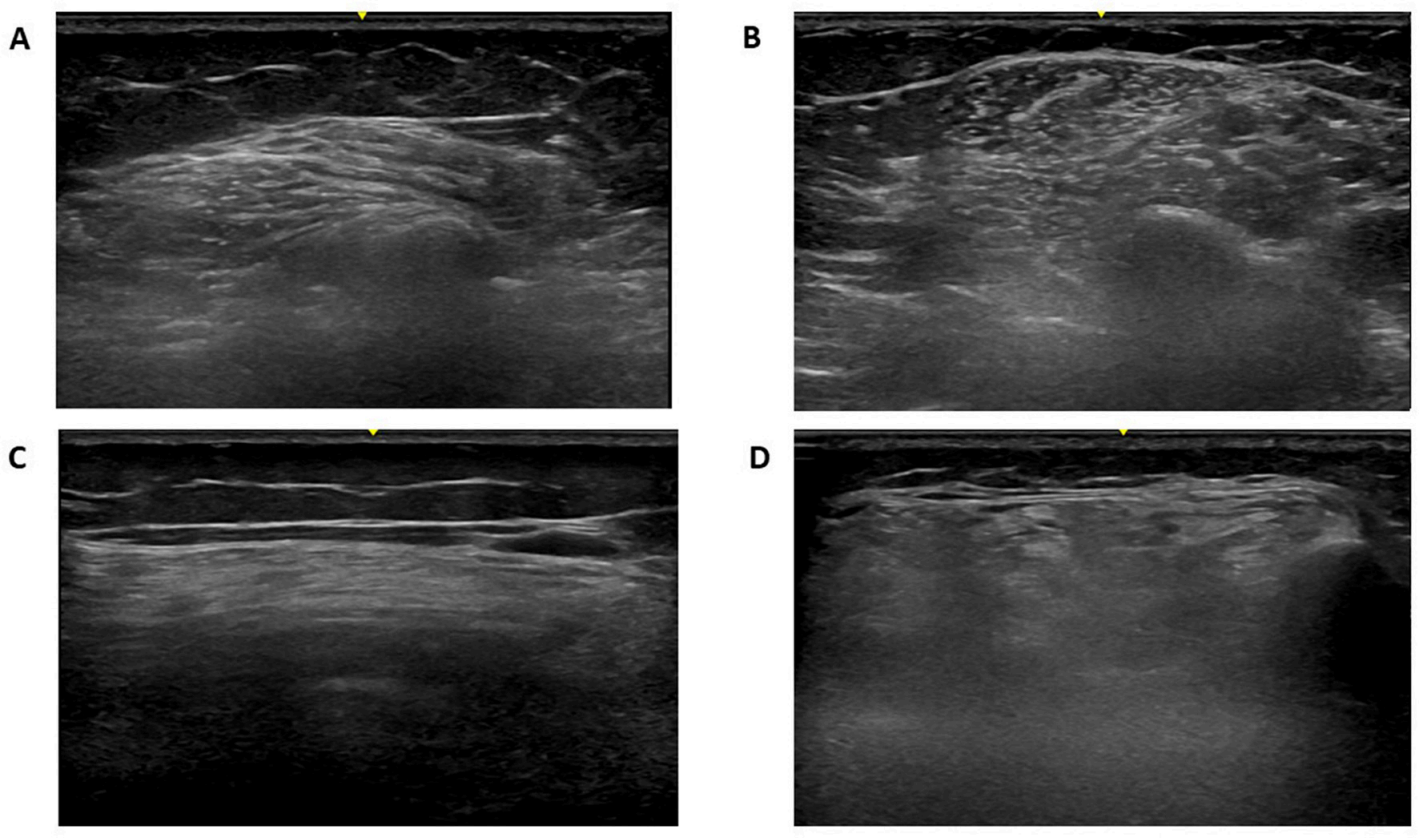
Muscle ultrasound of patient 5 with GFPT1-related congenital myasthenic syndrome demonstrating severe myopathic changes. Muscle ultrasound images from Patient 5 with GFPT1-related congenital myasthenic syndrome, demonstrating advanced myopathic changes graded by the modified Heckmatt scale. **(A)** Right deltoid (Grade 3) **(B)** Right biceps brachii (Grade 3); **(C)** Right vastus lateralis (Grade 4) **(D)** Right tibialis anterior (Grade 4).

In all patients, muscle ultrasound revealed increased echogenicity suggestive of myopathic involvement, with varying degrees of severity (see Table 3, Figure 3). The vastus lateralis and tibialis anterior muscles most frequently showed higher Heckmatt scores (grades 3–4), consistent with more advanced structural changes in these regions. Patient 5 (see Figure 3). demonstrated the most severe and generalized muscle involvement (grade 4 in three muscles), while patient 1 had milder findings (mostly grade 2). The deltoid and biceps muscles generally showed milder involvement (grades 2–3). Ultrasound evaluation revealed consistent involvement of the tibialis anterior muscle across the cohort. In most patients, the tibialis anterior muscle displayed a degree of structural alteration comparable to the vastus lateralis, and in two cases (patients 1 and 4) the distal muscle was even more severely affected than proximal thigh muscles.

**Table 3:** Muscle ultrasound echogenicity assessed by the modified Heckmatt scale in patients with GFPT1-CMS.

**Patient**	**Upper limb muscles**		**Lower limb muscles**			**Mean HS**
	**Deltoid**	**Biceps**	**Vastus lateralis**	**Tibialis anterior**	**Gastrocnemius medialis**	
1	2	2	2	3	2	2.2
2	3	2	3	3	2	2.6
3	3	3	4	4	3	3.4
4	2	2	2	3	2	2.2
5	3	3	4	4	4	3.6

B-mode muscle ultrasound echogenicity across five muscles: deltoid, biceps brachii (upper limbs), vastus lateralis, tibialis anterior, and gastrocnemius medialis (lower limbs). Muscle involvement was graded using the modified Heckmatt scale (Grade 1 = normal echogenicity; Grade 2 = mildly increased; Grade 3 = moderately increased with partial loss of architecture; Grade 4 = severely increased echogenicity with loss of normal muscle architecture). The mean Heckmatt score (Mean HS) was calculated for each patient as the average echogenicity across the five muscles. Higher scores indicate more pronounced structural muscle involvement. Patients with higher MGFA severity scores (e.g., IVa) tended to show higher mean Heckmatt scores.

Mean Heckmatt scores ranged from 2.2 to 3.6, indicating varying degrees of muscle echogenicity and structural damage (see Table 3). A strong positive correlation was found between MGFA score and mean Heckmatt echogenicity score (Spearman ρ = 0.918, *p* = 0.028) (Table 4), suggesting that clinical severity of weakness was aligned with increased structural muscle abnormalities on ultrasound. Specifically, the patient classified as MGFA IVa (Patient 5), who exhibited the most severe phenotype, including early loss of ambulation, also demonstrated the highest mean echogenicity score (Figure 3). Muscle-specific analyses showed no significant correlations between RNS decrements and ultrasound echogenicity: deltoid 39.0–45.7% (mean 43.6%, HS 2–3; *p* = 1.00) and tibialis anterior 21.7–61.7% (mean 36.5%, HS 3–4; *p* = 0.23).

**Table 4:** Correlation between MGFA clinical severity and mean muscle echogenicity in patients with GFPT1-CMS.

**Variables**	**Spearman r**	***p*-value**
MGFA severity score × mean Heckmatt score	0.918	**0.028**

Spearman correlation between MGFA clinical severity score and mean muscle echogenicity (assessed by the modified Heckmatt scale) in five patients with GFPT1-related congenital myasthenic syndrome (GFPT1-CMS). A strong and statistically significant positive correlation was found, indicating that higher clinical severity was associated with greater structural muscle involvement on ultrasound. Bold values indicate statistically significant results (*p* < 0.05).

## 4 Discussion

This study revealed that *GFPT1*-related CMS exhibits a broad and heterogeneous clinical spectrum, with notable intrafamilial phenotypic variability, including significant differences in presentations between twins. Consistent findings of our cohort included a limb-girdle pattern of weakness with distal lower limb involvement, particularly foot dorsiflexion (tibialis anterior) weakness, early-onset frequent falls, and a uniformly positive response to acetylcholinesterase inhibitors. One patient (Case 5) presented unique features not previously described in *GFPT1*-CMS, including intellectual disability and epilepsy, suggesting that central nervous system involvement may also occur in this condition.

*GFPT1*-related CMS should be considered in the differential diagnosis of any limb-girdle weakness phenotype, even in the absence of family history or clear fatigability developing in the first decade of life (18, 19). Additionally, less frequent phenotypic features may include flat feet and Achilles tendon retractions (20), axial weakness involving the neck flexors (20) and abdominal muscles, as well as bilateral scapular winging (20). Some patients may also present with dysmorphic features such as a slender neck, thin upper lip, prominent forehead, and hyperextensible joints (19). Psychomotor delay has been reported in only one isolated case (20). Extraocular involvement is uncommon but has been recently described, including eyelid ptosis and mild ophthalmoparesis in a Chinese cohort (21). There are also reports of asymptomatic leukoencephalopathy affecting the deep cerebral white matter and the corpus callosum (22). To our knowledge, we present the first patient with epilepsy associated with *GFPT1*-CMS, possibly expanding the neurological spectrum of the disease.

The *GFPT1* c.41G>A (p.Arg14Gln) variant was first clinically characterized in a study by Mensch et al., where a case series of three siblings with biallelic *GFPT1* variants was reported, with pathogenicity further confirmed through histological and biochemical analyses (13). This study identified compound heterozygous variants in *GFPT1*, c.41G>A (p.Arg14Gln) and c.1265_1268del (p.Phe422TrpfsTer26). The clinical presentation was remarkable for progressive proximal weakness, respiratory involvement, and ultimately a lethal course in adulthood (13). Notably, there was considerable intrafamilial variability in the severity of symptoms. The affected siblings displayed strikingly different trajectories: one brother had severe early-onset weakness, never achieved independent walking, and died at 49 years from respiratory failure; another brother showed childhood-onset but more slowly progressive limb-girdle weakness, attained independent ambulation, and lived until 40 years before ventilatory failure; while the youngest sister presented with an intermediate course, achieving independent ambulation at 6 years, later progressing to wheelchair dependence by adolescence, with bulbar and respiratory involvement, and died at 18 years. Together, these observations highlight marked intrafamilial heterogeneity, ranging from profound infantile weakness with early functional loss to a more indolent course with delayed loss of ambulation, underscoring the variable natural history even within a single family (13). The clinical spectrum associated with the *GFPT1* c.41G>A (p.Arg14Gln) variant shows both overlapping and divergent features when comparing our cohort to the previously reported family by Mensch et al. (13). In both studies, the variant was associated with a limb-girdle pattern of weakness and considerable intrafamilial variability. However, important differences were observed. In the family described by Mensch et al., the variant was present in compound heterozygosity with c.1265_1268del (p.Phe422TrpfsTer26) (13), and the clinical course was uniformly severe, with early loss of ambulation, progressive respiratory involvement, and premature death between 18 and 49 years of age. In contrast, all patients in our series carried the p.Arg14Gln variant in homozygosis and exhibited preserved respiratory function and absence of bulbar or ocular involvement (13). Although variability in severity was also present, only one patient, who presented with neonatal onset, developed early loss of ambulation and displayed additional manifestations not previously described in *GFPT1*-CMS, including epilepsy, psychiatric symptoms, and intellectual disability. These findings suggest that the p.Arg14Gln variant might lead to a wide phenotypic continuum, ranging from severe, life-limiting disease to milder forms with long-term survival and preserved ambulation.

All five patients carried the same homozygous c.41G>A (p.Arg14Gln) variant in *GFPT1*. The occurrence of this rare homozygous variant in two apparently unrelated families is highly unusual and raises the possibility of a shared genetic background. Both families originate from the same geographic region, residing in neighboring cities approximately 10 km apart, which further supports the hypothesis of a potential founder effect. However, haplotype analysis could not be performed.

Some CMS subtypes—such as those associated with *GFPT1, DOK7, GMPPB, DPAGT1, ALG2, and ALG14* genes—are known to present overlap with myopathic changes on muscle biopsy (9, 20, 23–25). This concept supports the proposition that distinct CMS subtypes may exhibit specific patterns on muscle imaging investigations, thereby facilitating differential diagnosis, guiding targeted genetic testing, and providing earlier therapeutic intervention (26). Few studies have evaluated muscle imaging in CMS (21, 26, 27). Among CMS subtypes, *GFPT1*-CMS has been associated with the most severe degree of fatty infiltration, as indicated by T1-weighted (T1W) sequences on magnetic resonance imaging (MRI), which reveal a diffuse, non-selective pattern of muscle involvement in the thigh (26). In contrast, MRI findings in a patient with *ALG14*-CMS—a disorder also caused by mutations in a glycosylation pathway gene—revealed only mild muscle changes despite a disease duration of 47 years (26). Our findings expand the literature on muscle imaging in *GFPT1*-related CMS. Although *GFPT1*-CMS has been classically characterized as a limb-girdle myasthenic syndrome, our findings indicate that distal muscles, particularly the tibialis anterior, can be equally or even more severely affected. Jiang et al. (27) showed two patients with a selective distribution of myopathic changes in MRI with diffuse involvement of the thigh with relative sparing of the adductor magnus and semimembranosus, along with leg compromise with medial gastrocnemius sparing (tibialis muscles in the anterior compartment of the leg being the most affected) (27) The observation that tibialis anterior involvement was as marked as the vastus lateralis in most patients in our cohort, and disproportionately severe in some, highlights a broader phenotypic spectrum than traditionally recognized (23). Distal weakness, corroborated by both ultrasound, RNS, and clinical examination, suggests that GFPT1-related pathology is not strictly confined to proximal musculature. The presence of dorsiflexion weakness should raise clinical suspicion for *GFPT1*-CMS, as observed in our cohort. Pyridostigmine was reported to benefit all patients in our cohort, particularly with subjective improvements in fatigue, speed, and agility. However, no patient experienced a substantial change in MGFA class after treatment. The underlying myopathic changes observed in *GFPT1*-related congenital myasthenic syndrome (27) might also contribute to the lack of significant MGFA improvement, further emphasizing that this scale may not be the most appropriate tool to capture treatment effects in this scenario. Future studies should incorporate more sensitive and quantitative outcome measures, such as the modified Quantitative Myasthenia Gravis (QMG) (28) score, which has been used in CMS (29) which may better reflect functional gains in this population.

In our study, a portable muscle ultrasound device identified significant structural changes, supporting its potential role as a non-invasive and accessible biomarker for detecting and monitoring muscle damage in CMS. Additionally, ultrasound may provide diagnostic value by revealing patterns of muscle involvement that can guide clinical investigation in the setting of a suspected CMS with a limb-girdle weakness pattern. Future comparative studies across different forms of limb-girdle CMS would be highly valuable to determine whether muscle change profiles differ among subtypes.

Our findings in Brazilian patients reinforce the importance of early and accurate diagnosis of *GFPT1*-CMS, especially in regions with limited access to genetic testing. Despite carrying the same pathogenic variant, patients showed a broad range of severity, highlighting potential modifying genetic or environmental factors. The integration of neurophysiological studies, muscle imaging, and clinical evaluation proved essential for detailed phenotyping and should be considered in CMS diagnostic protocols. Moreover, the identification of cognitive compromise and epilepsy in one patient suggests that *GFPT1*-related disease may, in rare cases, extend beyond the neuromuscular junction.

This study has some limitations, including a small sample size and the absence of longitudinal follow-up and standardized functional outcome measures. Formal scales for quantifying response to treatment were not employed, and muscle biopsy data were available in only one case (29). Nevertheless, this case series possibly expands the known phenotypic spectrum of *GFPT1*-CMS, notably reporting the first association with epilepsy, and demonstrates the utility of muscle ultrasound in characterizing structural muscle changes in these patients.

We conclude that *GFPT1*-CMS may present with variable severity of proximal and distal muscle weakness and, in some cases, be accompanied by additional features such as intellectual developmental disorder and epilepsy. Multimodal phenotyping, including muscle ultrasound and electrophysiology, adds diagnostic value and should be integrated into routine evaluation. Recognition of atypical features may prevent misdiagnosis and support the timely initiation of symptomatic therapy.

## Data Availability

The raw data are not publicly available due to privacy concerns but may be provided by the corresponding author upon reasonable request.

## References

[1] Engel AG Shen XM Selcen D Sine SM. Congenital myasthenic syndromes: pathogenesis, diagnosis, and treatment. Lancet Neurol. (2015) 14:420–34. doi: 10.1016/S1474-4422(14)70201-7 25792100 PMC4520251

[2] Ohno K Ohkawara B Shen XM Selcen D Engel AG. Clinical and pathologic features of congenital myasthenic syndromes caused by 35 genes—a comprehensive review. Int J Mol Sci Multidiscipl Digi Publish Inst. (2023) 24:3730. doi: 10.3390/ijms24043730 36835142 PMC9961056

[3] Theuriet J Masingue M Behin A Ferreiro A Bassez G Jaubert P . Congenital myasthenic syndromes in adults: clinical features, diagnosis and long-term prognosis. Brain. (2024) 147:3849–62. doi: 10.1093/brain/awae124 38696726 PMC11531845

[4] Finsterer J. Congenital myasthenic syndromes. Orphanet J Rare Dis. (2019) 14:57. doi: 10.1186/s13023-019-1025-5 30808424 PMC6390566

[5] Ziaadini B Ghaderi Yazdi B Dirandeh E Boostani R Karimi N Panahi A . DOK7 congenital myasthenic syndrome: case series and review of literature. BMC Neurol. (2024) 24:211. doi: 10.1186/s12883-024-03713-0 38907197 PMC11191154

[6] Natera-de Benito D Töpf A Vilchez JJ González-Quereda L Domínguez-Carral J Díaz-Manera J . Molecular characterization of congenital myasthenic syndromes in Spain. Neuromuscular Disord. (2017) 27:1087–98. doi: 10.1016/j.nmd.2017.08.003 29054425

[7] Mihaylova V Salih MAM Mukhtar MM Abuzeid HA El-Sadig SM Von Der Hagen M . Refinement of the clinical phenotype in musk-related congenital myasthenic syndromes. Neurology. (2009) 73:1926–8. doi: 10.1212/WNL.0b013e3181c3fce9 19949040

[8] Pinto MV Saw JL Milone M. Congenital vocal cord paralysis and late-onset limb-girdle weakness in MuSK–congenital myasthenic syndrome. Front Neurol. (2019) 10:1300. doi: 10.3389/fneur.2019.01300 31920924 PMC6934021

[9] Senderek J Müller JS Dusl M Strom TM Guergueltcheva V Diepolder I . Hexosamine biosynthetic pathway mutations cause neuromuscular transmission defect. Am J Hum Genet. (2011) 88:162–72. doi: 10.1016/j.ajhg.2011.01.008 21310273 PMC3035713

[10] Selcen D Shen XM Milone M Brengman J Ohno K Deymeer F . GFPT1-myasthenia. Neurology. (2013) 81:370–8. doi: 10.1212/WNL.0b013e31829c5e9c 23794683 PMC3772836

[11] Prior DE Ghosh PS. Congenital myasthenic syndrome from a single center: phenotypic and genotypic features. J Child Neurol. (2021) 36:610–7. doi: 10.1177/0883073820987755 33471587

[12] Spendiff S Lochmüller H Maselli RA. Congenital myasthenic syndromes. Int Rev Neurobiol. (2025) 182:253–74. doi: 10.1016/bs.irn.2025.04.025 40675739

[13] Mensch A Cordts I Scholle L Joshi PR Kleeberg K Emmer A . GFPT1-associated congenital myasthenic syndrome mimicking a glycogen storage disease – diagnostic pitfalls in myopathology solved by next-generation-sequencing. J Neuromuscul Dis. (2022) 9:533–41. doi: 10.3233/JND-220822 35694932

[14] Jaretzki A III Barohn RJ Ernstoff RM Kaminski HJ Keesey JC Penn AS . Views & reviews Myasthenia gravis recommendations for clinical research standards. Neurology. (2000) 55:16–23. doi: 10.1212/WNL.55.1.16 10891897

[15] Richards S Aziz N Bale S Bick D Das S Gastier-Foster J . Standards and guidelines for the interpretation of sequence variants: a joint consensus recommendation of the American College of Medical Genetics and Genomics and the Association for Molecular Pathology. Genet Med. (2015) 17:405–24. doi: 10.1038/gim.2015.30 25741868 PMC4544753

[16] Heckmatt JohnZ Dubowitz V Leeman S. Detection of pathological change in dystrophic muscle with b-scan ultrasound imaging. Lancet. (1980) 315:1389–90. doi: 10.1016/S0140-6736(80)92656-2 6104175

[17] Vill K Schessl J Teusch V Schroeder S Blaschek A Schoser B . Muscle ultrasound in classic infantile and adult Pompe disease: a useful screening tool in adults but not in infants. Neuromusc Disord. (2015) 25:120–6. doi: 10.1016/j.nmd.2014.09.016 25455803

[18] Maselli RA Arredondo J Nguyen J Lara M Ng F Ngo M . Exome sequencing detection of two untranslated GFPT1 mutations in a family with limb-girdle myasthenia. Clin Genet. (2014) 85:166–71. doi: 10.1111/cge.12118 23488891

[19] Vallepu SB Dhamija K Rajan GK Panchal T Saran RK Roshan S. Phenotypic variability in congenital myasthenic syndrome with GFPT1 mutation. Acta Neurol Belg. (2025) 125:209–13. doi: 10.1007/s13760-024-02694-8 39602055

[20] Bauché S Vellieux G Sternberg D Fontenille MJ De Bruyckere E Davoine CS . Mutations in GFPT1-related congenital myasthenic syndromes are associated with synaptic morphological defects and underlie a tubular aggregate myopathy with synaptopathy. J Neurol. (2017) 264:1791–803. doi: 10.1007/s00415-017-8569-x 28712002

[21] Zhang J Chen X Yan C Gu X Zhu W Cao X . A cohort of GFPT1 related congenital myasthenic syndrome in China: high frequency of c331 c &gt; t variant. Orphanet J Rare Dis. (2025) 20:259. doi: 10.1186/s13023-025-03823-z 40442802 PMC12124097

[22] Helman G Sharma S Crawford J Patra B Jain P Bent SJ . Leukoencephalopathy due to variants in GFPT1- associated congenital myasthenic syndrome. Neurology. (2019) 92:e587–93. doi: 10.1212/WNL.0000000000006886 30635494

[23] Nicolau S Kao JC Liewluck T. Trouble at the junction: when myopathy and myasthenia overlap. Muscle Nerve. (2019) 60:648–57. doi: 10.1002/mus.26676 31449669

[24] Huh SY Kim HS Jang HJ Park YE Kim DS. Limb-girdle myasthenia with tubular aggregates associated with novel GFPT1 mutations. Muscle Nerve. (2012) 46:600–4. doi: 10.1002/mus.23451 22987706

[25] Muñoz-García MI Guerrero-Molina MP de Fuenmayor-Fernández de la Hoz CP Bermejo-Guerrero L Arteche-López A Hernández-Laín A . Delayed diagnosis of congenital myasthenic syndromes erroneously interpreted as mitochondrial myopathies. J Clin Med. (2023) 12:3308. doi: 10.3390/jcm12093308 37176748 PMC10179722

[26] Finlayson S Morrow JM Rodriguez Cruz PM Sinclair CDJ Fischmann A Thornton JS . Muscle magnetic resonance imaging in congenital myasthenic syndromes. Muscle Nerve. (2016) 54:211–9. doi: 10.1002/mus.25035 26789134 PMC4982021

[27] Jiang K Zheng Y Lin J Wu X Yu Y Zhu M . Diverse myopathological features in the congenital myasthenia syndrome with GFPT1 mutation. Brain Behav. (2022) 12:e2469. doi: 10.1002/brb3.2469 34978387 PMC8865156

[28] Barohn RJ McIntire D Herbelin L Wolfe Gi Nations S Bryan WW. Reliability testing of the quantitative myasthenia gravis score. Ann N Y Acad Sci. (1998) 841:769–72. doi: 10.1111/j.1749-6632.1998.tb11015.x 9668327

[29] Kastreva K Chamova T Blagoeva S Bichev S Mihaylova V Meyer S . Characterization of clinical phenotypes in congenital myasthenic syndrome associated with the c.1327delG frameshift mutation in chrne encoding the acetylcholine receptor epsilon subunit. J Neuromuscul Dis. (2024) 11:1011–20. doi: 10.3233/JND-230235 38995797 PMC11380250

